# Signal detection on spontaneous reports of adverse events following immunisation: a comparison of the performance of a disproportionality-based algorithm and a time-to-onset-based algorithm

**DOI:** 10.1002/pds.3502

**Published:** 2013-09-09

**Authors:** Lionel van Holle, Vincent Bauchau

**Affiliations:** Vaccine Safety Research Group (VSRG), Vaccines Clinical Safety & Pharmacovigilance (VCSP), GlaxoSmithKline VaccinesWavre, Belgium

**Keywords:** vaccine safety, signal detection, pharmacovigilance, disproportionality, time-to-onset, Kolmogorov-Smirnov, pharmacoepidemiology

## Abstract

**Purpose:**

Disproportionality methods measure how unexpected the observed number of adverse events is. Time-to-onset (TTO) methods measure how unexpected the TTO distribution of a vaccine-event pair is compared with what is expected from other vaccines and events. Our purpose is to compare the performance associated with each method.

**Methods:**

For the disproportionality algorithms, we defined 336 combinations of stratification factors (sex, age, region and year) and threshold values of the multi-item gamma Poisson shrinker (MGPS). For the TTO algorithms, we defined 18 combinations of significance level and time windows. We used spontaneous reports of adverse events recorded for eight vaccines. The vaccine product labels were used as proxies for true safety signals. Algorithms were ranked according to their positive predictive value (PPV) for each vaccine separately; amedian rank was attributed to each algorithm across vaccines.

**Results:**

The algorithm with the highest median rank was based on TTO with a significance level of 0.01 and a time window of 60 days after immunisation. It had an overall PPV 2.5 times higher than for the highest-ranked MGPS algorithm, 16^th^ rank overall, which was fully stratified and had a threshold value of 0.8. A TTO algorithm with roughly the same sensitivity as the highest-ranked MGPS had better specificity but longer time-to-detection.

**Conclusions:**

Within the scope of this study, the majority of the TTO algorithms presented a higher PPV than for any MGPS algorithm. Considering the complementarity of TTO and disproportionality methods, a signal detection strategy combining them merits further investigation.

## INTRODUCTION

Suspected adverse reactions following immunisation with marketed vaccines are reported to a spontaneous report system allowing continuous monitoring to detect new safety signals. These reports, coming from sources including regulatory authorities, health care professionals and consumers, are related to the real-life, post-licensure use of these vaccines. Those related to GlaxoSmithKline (GSK) vaccines are stored in the company's safety database Operating Company Event Accession and Notification System (OCEANS).

On 1 February 2010, OCEANS contained 147 015 spontaneous reports of 28 425 distinct vaccine-event pairs, involving 45 distinct GSK vaccines suspected and 4331 distinct MedDRA‡ preferred terms. These reports dated from 1987 to 2010.

The most frequent methods for analysing spontaneous reports in pharmacovigilance are numerator-based, disproportionality analyses (DPA) 1–6 being the most widely used. These methods aim to overcome the lack of reliable estimates of the exposed population. They focus on estimating the strength of association between a product and an event.

The multi-item gamma Poisson shrinker (MGPS) 2,7,8 is an empirical Bayes data mining algorithm for DPA. It uses information for all products and all events from a given safety database to compute an empirical Bayes geometric mean (EBGM) for each observed vaccine-event combination. EBGM values are adjusted estimates of relative reporting ratios (observed reporting rate/expected reporting rate) after Bayesian ‘shrinkage’. An EBGM value of 5 is interpreted to mean that a vaccine-event combination has been reported at least 5 times as frequently as would be expected if reports involving the vaccine and reports of the event were independent. The MGPS also computes the two-sided 90% credibility interval (CI: EB05, EB95) for each EBGM value. It offers the opportunity of internal stratification using a Mantel-Haenszel approach 7 for reducing the chance of spurious associations occurring because of confounding factors.^7^,^9^

We recently demonstrated that these vaccine-event pairs could also be routinely screened for unexpected time-to-onset (TTO) distribution with a two-sample Kolmogorov-Smirnov test.^10^ In this previous proof-of-concept study, a TTO signal was flagged if the TTO distribution of a vaccine-event pair differed significantly (at a 0.05 alpha level) from the expected TTO distribution of the [other vaccines]-[event-of-interest] and [vaccine-of-interest]-[other events] pairs within a time window of 30 days after immunisation. The proof-of-concept study results showed higher sensitivity and/or specificity associated with the TTO signal detection than with the MGPS stratified by sex, age, region and year of reporting, which flagged vaccine-event pairs as a signal by using a cut-off value of 2 on the EB05. The theoretical and practical complementarity of DPA and TTO signal detection methods was highlighted.

The aim of this paper is two-fold. Firstly, we compare the performance of the two methods by taking into consideration the parameters that could play a role in their respective performance. Two parameters were selected for the TTO signal detection: the alpha level and the time window. A previous evaluation of the performance of MGPS using the same data set showed that the set of stratification factors and, especially, the cut-off value influenced MGPS performance directly 11. Secondly, we provide follow-up to this previous publication and to the original proof-of-concept study of the TTO signal detection method.^10^

## METHODS

### Multi-item gamma Poisson shrinkage

A total of 336 different combinations of stratification factors ((S)ex, (A)ge, (R)egion and (Y)ear) and cut-off values for the MGPS usage were assessed (in a similar manner to that used in 11); 16 combinations of stratification factors (S, A, R, Y, SA, SR, SY, AR, AY, RY, SAR, SAY, ARY, SRY, SARY and (U)nstratified), each with 21 different cut-off values for the EB05 (from 0 to 4, incrementing by 0.2). Each MGPS was labelled as ‘threshold’-‘stratification’ (e.g. 2-SARY).

Each of these 336 MGPS algorithms was run against the entire GSK vaccines safety database frozen on February 2010 by using Empirica Signal (Oracle Corporation, Reading, UK).

### Time-to-onset signal detection

Time-to-onset signal detection is a non-parametric data mining algorithm for detecting vaccine-event pairs presenting TTO distributions that differ significantly from:

– the TTO distribution of the same vaccine but with other events reported (‘between events’)

and

– the TTO distribution of the same event but reported after administration of other vaccines (‘between vaccines’)

at a given significance alpha level and within a given time window.^10^ The test statistic is the two-sample Kolmogorov-Smirnov, 13 which is sensitive to any differences in the distribution from which the two samples were drawn, in terms of location, dispersion or skewness.

The TTO signal detection method aims at detecting patterns of TTO that deviate from the overall pattern of reported TTO assuming to be mainly driven by reporting biases and noise.

Eighteen different combinations of alpha levels (0.01, 0.05, 0.10, 0.20, 0.50 and 0.99) and time windows (30, 60 and 90 days) for TTO signal detection (SD) were investigated. Each TTO SD algorithm was labelled TTO-‘alpha level’-‘length of time window’.

### Data selection for comparing multi-item gamma Poisson shrinker and time-to-onset signal detection performance

For practical reasons, the evaluation was restricted to eight different vaccines (as done previously 11): *Rotarix*™ (live paediatric), *Engerix*™ (inactivated for adults), *Cervarix*® (inactivated for female adolescents), *Fluarix*™ (inactivated for adults), *Infanrix*™ (inactivated paediatric), *Infanrix*™ Hib (inactivated paediatric), *Havrix*™ (inactivated for adults) and *Twinrix*™ (inactivated for adults). These vaccines were selected for their heterogeneity of indications and their overall volume of reports. The characteristics of case reports are summarised for each vaccine in Table 1. This sample of vaccines can be considered as representative of the entire spontaneous report database at GSK vaccines, as it represents more than half of the reports in the database and shows diversity in vaccine characteristics.

**Table 1:** Demographic and secular characteristics of the eight vaccines under study in the spontaneous report database Operating Company Event Accession and Notification System

Vaccine	Age (years); Median (Q1,Q3)	Female (%)	Year of reporting; Median (Q1,Q3)	Number (%) of spont. reports	Number of countries
*Engerix*™	31.0 (18.0,43.0)	64.2	1999 (1993,2005)	34 347 (23.4%)	92
*Havrix*™	23.0 (11.0,40.0)	57.8	2004 (1998,2007)	9066 (6.2%)	58
*Cervarix*®	15.0 (12.0,17.0)	99.5	2009 (2008,2009)	3437 (2.3%)	63
*Infanrix*™	5.0 ( 1.5,10.0)	45.5	2006 (2003,2007)	9732 (6.6%)	59
*Infanrix*™ Hib	1.5 ( 0.8,1.9)	42.5	2002 (1999,2003)	1027 (0.7%)	21
*Rotarix*™	0.3 ( 0.2,0.6)	46.3	2008 (2007,2009)	2800 (1.9%)	73
*Fluarix*™	41.0 (19.0,60.0)	60.0	2005 (2002,2007)	6864 (4.7%)	69
*Twinrix*™	31.0 (19.0,45.0)	57.6	2006 (2003,2008)	9836 (6.7%)	51

For both the MGPS and the TTO SD method, the background comprised all vaccines (except the one of interest for the ‘between vaccines’ component of the TTO SD) in the GSK vaccines database and was not restricted to the eight vaccines described previously.

The proportion of vaccine-event pairs with a TTO between 0 and 90 days varied between 36.4% and 78.9% (Table 2). For each vaccine, variable proportions of vaccine-event pairs may have missing time-to-onset information. The proportion of vaccine-event pairs with TTO larger than 90 days also varies widely between vaccines (Table 2), mainly because of the differences in reporting rates of lack of efficacy events between vaccines.

**Table 2:** Time-to-onset characteristics of the eight vaccines under study in the spontaneous report database Operating Company Event Accession and Notification System

Vaccine	Number of vaccine-event pairs	% with missing TTO	% with TTO in [0,30] days	% with TTO in [0,60] days	% with TTO in [0,90] days	% with TTO > 90 days
*Engerix*™	119 440	51.9%	32.9%	35.3%	36.4%	11.6%
*Havrix*™	21 705	39.4%	52.7%	54.7%	55.7%	4.9%
*Cervarix*®	10 625	22.0%	75.0%	76.1%	76.6%	1.4%
*Infanrix*^*T*M^	22 507	17.9%	78.2%	78.6%	78.9%	3.2%
*Infanrix*™ Hib	3176	12.2%	51.3%	52.2%	52.8%	35.0%
*Rotarix*™	8019	15.3%	54.7%	58.7%	62.1%	22.6%
*Fluarix*™	19 028	32.5%	62.7%	64.4%	65.2%	2.3%
*Twinrix*™	29 130	33.0%	51.7%	54.8%	56.0%	11.0%

TTO= time-to-onset.

### The gold standard assumption

To assess the performance of each algorithm, a gold standard is needed to identify all events that are ‘truly causally’ related to the vaccine. This set of events is unknown but can be approximated by events listed in the Global Product Information (GPI). For each of the eight vaccines, each adverse reaction listed within the core company safety information of the GPI was mapped to one or more synonymous or medically equivalent MedDRA preferred terms (PTs). These MedDRA PTs were used as a proxy of the set of true signals.^8^,^10^

### The measurement of performance

#### 1) Overall performance

Using the gold standard definition and signal detection scores, we classified each reported vaccine-MedDRA PT pair as true positive (TP), false positive (FP), true negative (TN) or false negative (FN).

As described previously,^11^ we considered the positive predictive value (PPV = TP/(TP + FP)) rank as the main measure of performance. Note that ranks were defined on the basis of the descending order of the PPV (rank 1 is referred to as the ‘highest’ rank and corresponds to the highest PPV). The negative predictive value (NPV = TN/(TN + FN)), sensitivity, specificity, number of TP, FP, TN and FN were considered as secondary measures.

The median rank of the PPV associated with each parameter was computed with its standard deviation of the rank across all vaccines to estimate robustness of the performance.

Receiver operating characteristic (ROC) curves 12 displaying the relation between sensitivity and specificity were produced for the three TTO algorithms (with time windows of 30, 60 and 90 days after immunisation) by varying the *p*-value cut-off and for the 16 MGPS algorithms (with different combinations of stratification factors) by varying the cut-off on the EB05.

#### 2) The timing of detection

Another aspect in the performance of a signal detection algorithm is the timing of the detection. The detection dates of TP signals flagged by the highest PPV-ranked MGPS algorithm and by the TTO algorithm detecting approximately the same number of TP signals were compared to determine which algorithm detects TP signals more rapidly (in terms of minimal number of spontaneous reports). For each of the two signal detection algorithms, we compared the number of spontaneous reports present in OCEANS at the time of first detection of a TP signal. We also compared the number of spontaneous reports actually used by each method at the time of first detection. Indeed, the spontaneous reports without TTO information in the time window of interest were not considered as ‘used’ by the TTO signal detection algorithm whereas they were considered as ‘used’ by the MGPS algorithm.

### RESULTS

#### 1) Overall performance

The algorithm with the highest median PPV rank was a TTO algorithm with an alpha level of 0.01 for the Kolmogorov-Smirnov tests and a time window of 60 days after immunisation (Table 3). The highest-ranked MGPS algorithm was ranked only 16^th^ and was stratified by sex, age, region and year with a cut-off value of 0.8 (0.8-SARY), consistent with previous results where the range of investigated thresholds for the EB05 was between 0 and 2.^11^ The highest-ranked TTO algorithm had an overall PPV 2.5 times higher than the highest-ranked MGPS algorithm (0.8-SARY). The highest-ranked MGPS algorithm with an EB05 threshold higher than 2 was ranked only 75^th^ (2.4-SRY).

**Table 3:** Median positive predictive value rank across vaccines, overall positive predictive value, negative predictive value, numbers of true positives, false positives, true negatives, false negatives, sensitivity and specificity associated with the multi-item gamma Poisson shrinker and time-to-onset algorithms ordered

Algorithm	Median rank	PPV	NPV	TP	FP	TN	fn	Sensitivity	Specificity
TTO-01-60	1.50	0.592	0.924	77	53	8791	722	0.09637	0.994
TTO-01-30	3.00	0.630	0.923	68	40	8804	731	0.08511	0.995
TTO-01-90	3.00	0.590	0.924	79	55	8789	720	0.09887	0.994
TTO-05-30	5.00	0.517	0.927	107	100	8744	692	0.13392	0.989
TTO-05-60	5.25	0.487	0.927	116	122	8722	683	0.14518	0.986
TTO-05-90	7.75	0.461	0.927	117	137	8707	682	0.14643	0.985
TTO-10-60	8.50	0.410	0.929	134	193	8651	665	0.16771	0.978
TTO-10-30	9.00	0.435	0.928	124	161	8683	675	0.15519	0.982
TTO-10-90	9.00	0.399	0.929	137	206	8638	662	0.19274	0.977
TTO-20-30	10.00	0.364	0.930	154	269	8575	645	0.19274	0.970
TTO-20-60	12.00	0.340	0.931	169	328	8516	630	0.21151	0.963
TTO-20-90	12.00	0.340	0.932	177	343	8501	622	0.22153	0.961
TTO-50-60	14.75	0.261	0.937	254	721	8123	545	0.31790	0.918
TTO-50-90	15.25	0.256	0.938	262	763	8081	537	0.32791	0.914
TTO-50-30	16.00	0.265	0.936	239	663	8181	560	0.29912	0.925
0.8-SARY	19.50	0.204	0.930	195	763	8081	604	0.24406	0.914
0.8-ARY	23.50	0.199	0.930	195	784	8060	604	0.24406	0.911
0.8-SAY	34.50	0.182	0.919	185	829	8015	614	0.23154	0.906
…	…	…	…	…	…	…	…	…	…
2.4-SARY	118	0.185	0.918	23	101	8743	776	0.029	0.989
…	…	…	…	…	…	…	…	…	…
2-SARY	253	0.131	0.917	8	53	8791	791	0.01	0.994
…	…	…	…	…	…	…	…	…	…
3.4-ARY	28.25	0.05	0.917	1	19	8825	798	0.001	0.998

PPV= positive predictive value; NPV= negative predictive value; TP= true positive; FP= false positive; TN= true negative; FN= false negative.

**Figure 1:**
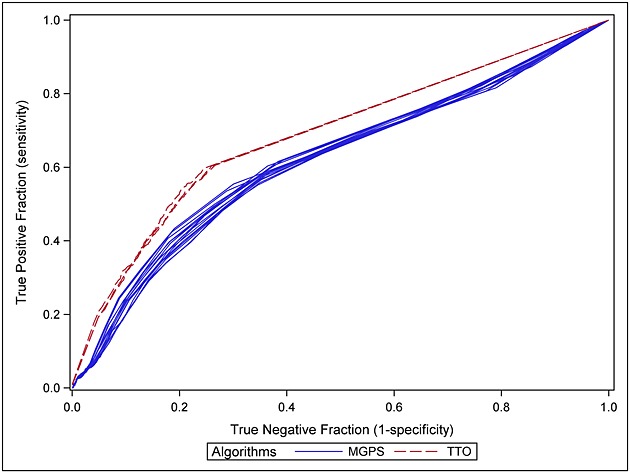
Receiver operating characteristic curves associated to the three time-to-onset algorithms based on time window length of 30, 60 and 90 days after immunisation (red dotted line) and the 16 choices of stratification of the multi-item gamma Poisson shrinker (blue solid line)

Receiver operating characteristic plots 12 were generated for the three TTO algorithms (with time windows of 30, 60 and 90 days after immunisation) by varying the *p*-value threshold from 0 to 1 by 0.01 and for each MGPS set of stratification factors by varying the EB05 threshold from 0 to 10 by 0.1 (Figure 1). The ROC curves associated to TTO algorithms are above all MGPS ROC curves, whatever the choice of stratifications.

**Figure 2:**
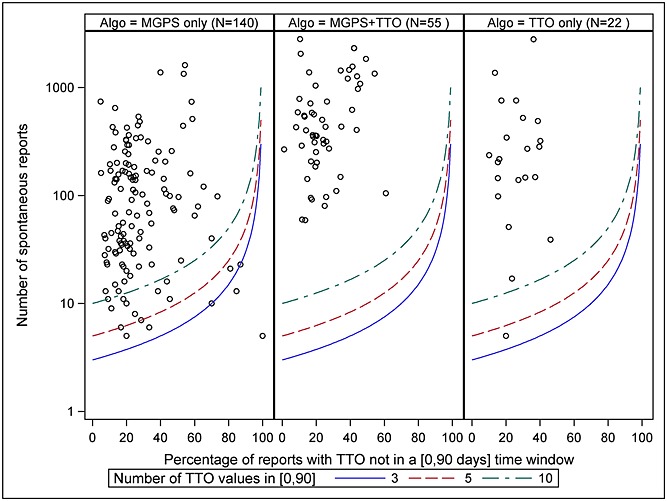
Characteristics of the true positive signals detected by the highest ranking algorithms (0.8-SARY and TTO-01-60)

The entire set of TP signals detected by the highest ranking MGPS alone, the highest ranking TTO alone, or by both algorithms is shown in Figure 2 along with the corresponding number of spontaneous reports and its proportion of reports with time-to-onset values outside of the [0, 90] days interval (TTO missing or beyond 90 days). Each point represents a vaccine-event pair. Each curve represents a given number of spontaneous reports with TTO within the [0, 90] days interval.

The TP signals detected by the highest ranking MGPS 0.8-SARY are well distributed in space [number of spontaneous reports]-[% of reports with TTO outside of the (0,90) time window]. On the other hand, the highest ranking TTO signal detection algorithm TTO-01-60 did not detect TP signals from a zone characterised by a high percentage of reports with time-to-onset values outside of the [0, 90] days interval or a low number of spontaneous reports. The highest ranking MGPS algorithm was able to detect some TP signals in this zone. A minimum of ten time-to-onset values in the interval [0, 90] days discriminates the zone where signals are systematically missed by the highest ranking TTO signal detection method characterised by highest PPV. The TP signals detected by both algorithms are characterised by a higher number of spontaneous reports.

**Figure 3:**
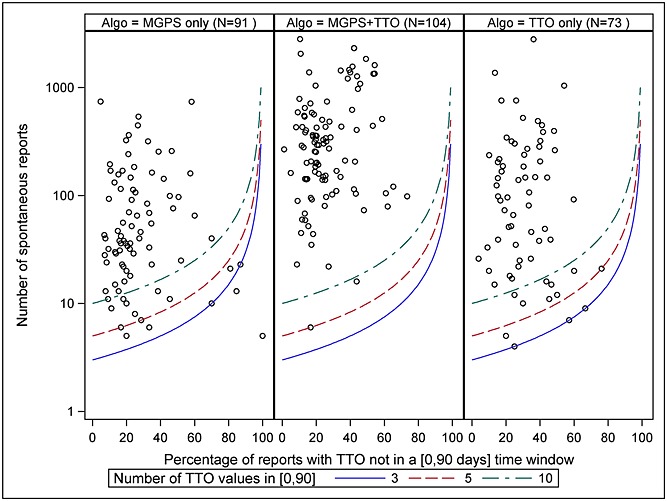
Characteristics of the true positive signals detected by the algorithms 0.8-SARY and TTO-20-90

The TTO-20-90 algorithm, which detected a similar number of TPs for fewer FPs than the highest-ranked MGPS algorithm 0.8-SARY (Table 3), can detect TP signals from three spontaneous reports with not missing TTO values in the [0, 90] days interval (Figure 3). Only two signals detected by the MGPS algorithm alone were below this limit.

#### 2) The timing of detection

The TTO-20-90 and 0.8-SARY algorithms detected approximately the same number of TP signals (177 and 195, respectively); 104 were detected by both.

**Figure 4:**
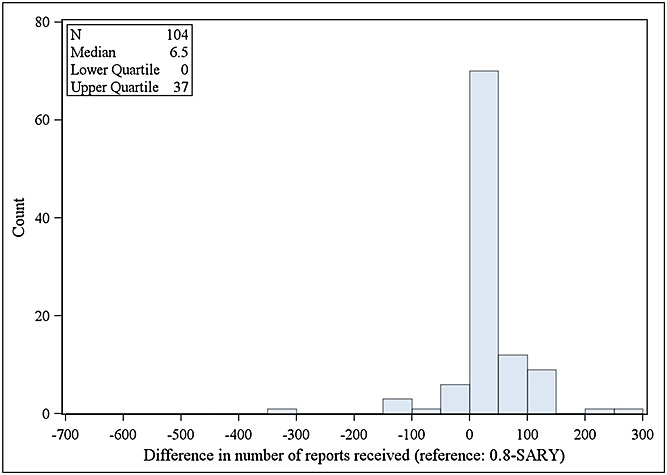
Difference in the minimal number of spontaneous reports received for first detection of true positive signals by TTO-20-90 and 0.8-SARY algorithms (positive numbers indicate that TTO-20-90 requires a higher minimal number of spontaneous reports in Operating Company Event Accession and Notification System)

**Figure 5:**
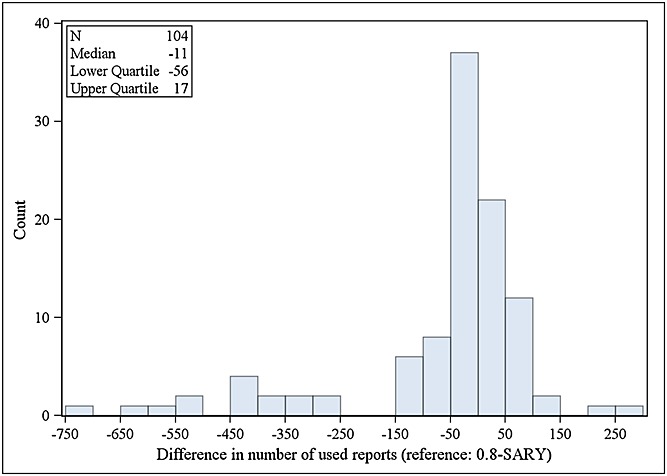
Difference in the minimal number of spontaneous reports actually used for first detection of true positive signals by TTO-20-90 and 0.8-SARY algorithms (positive numbers indicate that TTO-20-90 ‘uses’ a higher minimal number of reports)

The results of these two time-to-detection analyses showed that, on average, the algorithm 0.8-SARY needs less spontaneous reports (a median of 6.5 spontaneous reports less) than the algorithm TTO-20-90 for first detection of a TP signal (Figure 4). However, the signals found by TTO-20-90 are on average based on a smaller number of case reports than those used by 0.8-SARY (a median of 11 spontaneous reports less) (Figure 5).

### DISCUSSION

The highest-ranked TTO signal detection algorithm performs up to 2.5 times better, in terms of overall PPV, than the highest-ranked MGPS. This TTO signal detection algorithm, which uses an alpha level of 0.01 and a time window of 60 days, is also more specific than any MGPS algorithm and provides then a more manageable number of signals compared with the optimised MGPS fully stratified by sex, age, region and year with an EB05 cut-off of 0.8.^11^

When looking at secondary performance measures, this highest-ranked TTO SD algorithm was characterised by lower sensitivity than the highest-ranked MGPS. However, other TTO SD algorithms with higher alpha levels (such as TTO-20-90) provided similar sensitivity and numbers of TP signals than the highest-ranked MGPS algorithm but with higher specificity and a higher PPV.

The ROC curves highlighted that the performance of the TTO algorithms are above the performance of the MGPS algorithms whatever the choice of stratification factors and independently of a cut-off choice. However, for TTO algorithms, the sensitivity was truncated to 60%, and the only way to achieve 100% of sensitivity was to consider every reported vaccine-event pair as a TTO signal even if no TTO information in the time window under scrutiny was available.

In terms of time-to-detection, the algorithm 0.8-SARY needed on average less spontaneous reports than the algorithm TTO-20-90 for first detection of a TP signal. However, the signals found by TTO-20-90 are on average based on a smaller number of case reports than those used by 0.8-SARY as this last one requires all spontaneous report data and not only the subset of those with non-missing time-to-onset information within a 90 days period after immunisation. Consequently, the fact that 0.8-SARY detects on average faster than the TTO-20-90 algorithm can be attributed to the fact it uses all spontaneous reports. The better performance of 0.8-SARY over TTO-20-90 in terms of time-to-detection could be challenged in case the quality of the reporting of time-to-onset information increases. The time-to-detection was assessed based only on the subset of TP signals detected by both algorithms. However, not all vaccine-event pairs presenting a causal association have the potential for being detected by both 0.8-SARY and TTO-20-90. Indeed, some events could have no specific temporal relationships in the 90 days period after immunisation but could still be characterised by a number of observed cases higher than expected. That could happen for long-term events or when the TTO is systematically missing. On the other hand, some events could be characterised by only a very small excess of observed (reported) cases not enough to be detected by 0.8-SARY but detectable by TTO-20-90 in case all the excess cases are in a very narrow time within the 90 days period after immunisation Consequently, the measure of relative performance of 0.8-SARY and TTO-20-90 depends of the events labelled and the related products. It could favour one method over the other, making the generalisation to other products (like drugs for example) hard.

The overall performance was better for the TTO signal detection method than for the MGPS signal detection method. Although, the MGPS method used all vaccine-event pairs from OCEANS, the TTO signal detection method used only 55% of them. That could be explained by the fact that the TTO information may more closely predict causality than the strength of association. Indeed, a systematic review of the methods used for causality assessment of adverse drug reactions showed that the TTO was the most frequent criterion used to assess causality across different methods.^14^ The GPI used here as a proxy for gold standard may contain proportionally more events with unexpected TTOs because the inclusion of events in the GPI is mainly, if not completely, driven by causality assessments.

The empirical comparison of the performance of the MGPS and TTO algorithms on the eight vaccines under study showed the promising potential of the TTO signal detection method. However, some limitations have to be kept in mind. Indeed, the comparison of performance was retrospective and used the GPI as gold standard. As the reporting of known adverse events following immunisation is likely to differ from that of unknown safety risks, either in reporting rate or in time-to-onset distribution, the assessed performance of each algorithm to detect listed events may differ from the performance in detecting unknown safety risks.

Currently, the most widely used signal detection algorithms 1–6 are based solely on disproportionality, which provides an estimate of the strength of association by coping with some constraints typical of spontaneous report data (e.g. the lack of exposure data). These different signal detection algorithms focused solely on the strength of association, despite many other criteria playing a role in causality assessment,^14^ because it is the only causality criterion that could be quantified and generated at the scale of an entire safety database without requesting prior analysis by medical experts. TTO signal detection algorithms now offer the possibility to quantify the unexpectedness of the TTO distributions by coping with constraints typical of spontaneous report data (the reporting bias over time — the later the event occurs after immunisation, the lower the chance it has to be reported).

As stressed in the proof-of-concept study,^10^ both methods are complementary theoretically and in their limitations. The TTO signal detection method is based on TTO data, which are neglected by the MGPS and which are recognised to be an important criterion to assess possible causality during medical evaluation of individual case reports. There is also less of a need for a large-sized background using TTO than the MGPS. However, TTO signal detection can only be performed on spontaneous reports presenting time-to-onset values within the time window of interest. This excludes spontaneous reports for which TTO information is missing or occurs after the predefined time window. Additionally, TTO signals may be missed for vaccine-event pairs that have few reports with available TTO information. The MGPS requires adjustment as the reporting rates can differ strongly among strata defined by demographic or secular characteristics, but can be performed on uncommon or long-term adverse reactions.

Only flagging signals that are detected by both MGPS and TTO SD methods would result in a system with lower sensitivity and higher specificity than either individual method. Knowing that we would systematically lose the ability to detect uncommon and long-term events, we would *a priori* not consider this option as viable for a signal detection system. On the other hand, flagging signals that are detected by the MGPS or the TTO SD would result in a signal detection system with low specificity and high sensitivity. This may not be optimal considering the difference in performance between the TTO and MGPS signal detection algorithms.

Consequently, further methodological research is needed to build a signal detection algorithm that accounts for both causality criteria: the strength of association estimated by a disproportionality measure and the unexpectedness of the time-to-onset distribution estimated by the two-sample Kolmogorov-Smirnov tests. As summarised by Manfred Hauben,^15^ ‘Finding ways to integrate quality/data criteria related to individual causality assessment may have the potential for a quantum leap in mining high-grade ore from spontaneous reports’.

Any spontaneous reporter of an adverse event (such as a health care professional and so forth) should be made aware of the importance of reporting as precise and complete TTO information as possible through spontaneous reporting systems. The coding of the TTO information into the spontaneous safety database should reflect the level of precision provided by the reporter: the reported precision unit could be minutes, hours, days, weeks, months or even years. As demonstrated here, TTO data may be used not only for causality assessment at the case level but also for signal detection.

### CONCLUSIONS

For the eight vaccines under study, the majority of the TTO algorithms provided a higher proportion of TP signals than any MGPS algorithm.

Nevertheless, the TTO method is dependent on the quality of the TTO data, which depends on the safety database and the data provided by the reporter.

We suggest using both TTO and disproportionality methods in parallel to benefit from the greater ability of the TTO method to detect TP signals and avoid signals being missed (or delayed) when TTO data are of low quality. However, additional research is still needed to build the statistical framework to facilitate this parallel usage.

*ROTARIX*, *ENGERIX*, *FLUARIX*, *INFANRIX*, *HAVRIX* and *TWINRIX* are trademarks of the GSK group of companies.

*CERVARIX* is a registered trademark of the GSK group of companies.

### CONFLICT OF INTEREST

Both authors are employees of GSK vaccines.

**KEY POINTS**

- Disproportionality methods measure the strength of association between a vaccine and an event.
- TTO signal detection methods measure how unexpected the TTO distribution of a vaccine-event pair is.
- A comparison of the different parameterization choices of both methods highlighted the better performance, in terms of PPV, of the TTO signal detection method over the disproportionality method on the GSK vaccine spontaneous report data.
- Because of the complementarity of the two methods, a signal detection strategy combining both of them merits further investigation

## Footnotes

‡ Medical Dictionary for Regulatory Activities (MedDRA) is a clinically validated international medical terminology used by regulatory authorities and the regulated biopharmaceutical industry throughout the entire regulatory process, from pre-marketing to post-marketing activities, and for data entry, retrieval, evaluation and presentation.
